# Medication regimen complexity and its impact on medication adherence in patients with multimorbidity at a comprehensive specialized hospital in Ethiopia

**DOI:** 10.3389/fmed.2024.1369569

**Published:** 2024-05-27

**Authors:** Abebe Tarekegn Kassaw, Ashenafi Kibret Sendekie, Amare Minyihun, Begashaw Melaku Gebresillassie

**Affiliations:** ^1^Department of Pharmacy, College of Health Sciences, Woldia University, Woldia, Ethiopia; ^2^Department of Clinical Pharmacy, School of Pharmacy, College of Medicine and Health Sciences, University of Gondar, Gondar, Ethiopia; ^3^Department of Health Systems and Policy, Institute of Public Health, College of Medicine and Health Sciences, University of Gondar, Gondar, Ethiopia; ^4^School of Medicine and Public Health, The University of Newcastle, Newcastle, New South Wales, Australia

**Keywords:** medication regimen complexity, multimorbidity, medication adherence, chronic diseases, Ethiopia

## Abstract

**Background:**

Medication regimen complexity (MRC) is suspected to hinder medication adherence in patients with multiple illnesses. Despite this, the specific impact on Ethiopian patients with multimorbidity is unclear. This study assessed MRC and its impact on medication adherence in patients with multimorbidity.

**Methods:**

A hospital-based cross-sectional study was conducted on patients with multimorbidity who had been followed at the University of Gondar Comprehensive and Specialized Hospital (UoGCSH), Ethiopia, from May to July 2021. Medication complexity was measured using the validated Medication Regimen Complexity Index (MRCI) tool, and the Adherence in Chronic Diseases Scale (ACDS) was used to measure medication adherence. Pearson’s chi-square test was used to examine associations between MRCI levels and medication adherence. Ordinal logistic regression analysis was used to determine the impact of MRC and other associated variables on medication adherence. Statistical significance was determined using the adjusted odds ratio (AOR) at *p*-value <0.05 and its 95% confidence range.

**Results:**

Out of 422 eligible patients, 416 (98.6%) were included in the study. The majority of participants (57.2%) were classified as having a high MRCI score with a mean (±SD) score of 9.7 (±3.4). Nearly half of the patients (49.3%) had low medication adherence. Patients with medium (AOR = 0.43, 95% CI: 0.04, 0.72) and higher (AOR = 0.31, 95% CI: 0.07, 0.79) MRCI levels had lower odds of medication adherence. In addition, monthly income (AOR = 4.59, 95% CI: 2.14, 9.83), follow-up durations (AOR = 2.31, 95% CI: 1.09, 4.86), number of medications (AOR = 0.63, 95% CI: 0.41, 0.97), and Charlson comorbidity index (CCI) (AOR = 0.36, 95% CI: 0.16, 0.83) were significantly associated with medication adherence.

**Conclusion:**

Medication regimen complexity in patients with multimorbidity was found to be high and negatively impacted the levels of medication adherence. Healthcare providers and other stakeholders should seek interventions aimed at simplifying drug regimen complexity and improving adherence.

## Introduction

Patients with multiple chronic conditions could potentially have a complicated medication regimen that alters the level of medication adherence. Multimorbidity has been defined as the presence of two or more chronic conditions in one individual (1, 2). It affects 23–33% of adults, with the prevalence further rising to 75% or higher at the age of 70 (2–4). This trend will amplify as life expectancies rise and idviduals with 65 years of age or older are expected to live with more than four chronic conditions by 2035 (5). Patients with multimorbidity are associated with increased healthcare and social services utilization (6), increased healthcare costs (7), poorer clinical outcomes (8), and increased disability and mortality (3).

Patients with chronic diseases are usually taking medications for long-term management, and the benefits of medications in preventing or slowing the progression of the adverse consequences of these diseases are indisputable (9). However, patients with multimorbidity require complex clinical care, including treatment with multiple medications (polypharmacy) (10–12). Although medication adherence is a significant aspect of clinical practice and research, only around 50% of chronically ill individuals adhere to their medications even in developed countries, according to WHO data, and nonadherence was much higher in low- and middle-income countries (13). Guidelines also emphasize that medication adherence is the cornerstone for managing and preventing long-term complications in multimorbidity patients (14). However, poor medication adherence remains a barrier to effective treatment outcomes, particularly in the management of chronic conditions (15, 16). Non-adherence or failing to adhere to the prescribed instructions with prescribed medication regimens contributes to treatment failure, hospitalization risk, increased medical expense, and morbidity and mortality risks in patients on long-term therapeutic plans (16–18).

Different studies have documented that low adherence to medications is multifactorial and multidimensional and related to patients, physicians, and healthcare systems (11, 12). A study conducted on Ethiopian diabetes patients with comorbidity showed that the source of medication cost, monthly income, number of medications, and medical conditions were significantly correlated with the level of medication adherence (15). Other studies also revealed that personal beliefs, sociodemographic characteristics, medication regimen complexity, clinical characteristics, and the number of medical conditions are factors that influence medication adherence in patients with chronic diseases (19–21).

The complexity of medication regimens could significantly impact patients’ medication adherence, particularly in patients with multimorbidity who have been taking long-termtherapy (22, 23). The presence of high-complexity therapies is related to recent changes in the epidemiological profile and the wide availability of drugs on the market, as pharmacotherapy is the main therapeutic strategy to cure and control diseases (24). The complexity of the medication regimen is a term used to describe multiple characteristics of the drug regimen of a patient, beyond just the number of medications. This includes factors such as the number of doses per day, the number of units per dose, forms of dosage, and additional guidelines (25). MRCs are more likely to be error-prone and impact patient safety and quality of life (26). The complexity of a person’s medication regimen depends on the characteristics of the pharmacotherapy, such as the number of drugs consumed, their pharmaceutical forms, schedules, and doses, and additional instructions given by the physician (27). MRC is commonly involved in patients with long-term medication therapeutic needs, in particular (28–30). Studies have shown a negative association between increased MRC and nonadherence to prescribed medication regimens (28, 31–35).

There is an increasing number of patients with multimorbidity in Ethiopia, and most patients with chronic diseases have at least one comorbid chronic condition with a high number of prescribed medications (36–39). This could be attributed to the increasing complexity of medication regimens, which in turn impacts patients’ medication adherence. The burden could be significant particularly in the Ethiopian population with most of the patients having a lower level of health literacy (40, 41) and a higher level of misunderstanding of dosage regimen instructions (42). A part of this study also demonstrated that patients with higher regimen complexity scores had a lower quality of life (43). Despite some studies have shown that increasing of MRCI score negatively affects medication adherence only in patients with specific diseases, such as asthma (28) and diabetes (32), there is a paucity of evidence that showed the levels of MRC and its impact on medication adherence in patients with multimorbidity. As a result, assessing the extent of MRC and its impact on medication adherence in these high-risk patients is crucial to designing tailored interventions to maximize the treatment outcomes of patients living with multimorbidity. Therefore, this study assessed MRC and its impact on the levels of medication adherence in patients with multimorbidity having chronic follow-up at the University of Gondar Comprehensive and Specialized Hospital (UoGCSH), Ethiopia. In addition, this study also examined the association between other independent variables and medication adherence in patients with multimorbidity.

## Method and materials

### Study design, setting, and period

An institutional-based cross-sectional study was conducted on patients with multimorbidity who had chronic follow-ups at the University of Gondar Comprehensive Specialized Hospital (UoGCSH) from May 1 to July 30, 2021. The hospital is one of the largest teaching hospitals in the country and has served more than 9 million population in the catchment area.

### Study participants and eligibility criteria

The study population consisted of patients with multimorbidity who visited the UoGCSH chronic ambulatory clinic during the study period and fulfilled the inclusion criteria. To be included in the study, participants should be adults (18 years and older), have been diagnosed with at least two or more chronic conditions, and have been treated and followed up on for at least 3 months in the study area. Patients who had a serious mental illness and were unable to cooperate with the interview and sought emergency medical attention, those with incomplete medical records, and pregnant women because of special ethical requirements as a special population were excluded from the study.

### Sample size determination and sampling techniques

The sample size was determined by using a single population proportion formula:


$$n=Z^{2}p\;\left(1-p\right)/w^{2}$$

Where: *n*, the sample size required; w, the marginal error of 5% (*w* = 0.05); *Z*, the degree of accuracy required at a 95% level of significance (*Z* = 1.96); and *p* = 50% (0.5) is the level of regimen complexity. This is because no study was done previously in the study area. Finally, considering 10% of possible non-respondents, the final sample size was 422.

The study participants were selected using systematic random sampling techniques from eligible study populations. The projected number of patients with multimorbidity at two-month follow-up was 2,340, so *K* = 52 *45/422 = 5.5, approximately = 6. Initially, the first study participant was selected using simple random sampling as the starting point. Then, subsequently, participants were included in the study using this sampling interval, every six patients, through the coding of their medical records until sufficient samples were maintained.

### Operational definition of terms

#### Chronic diseases

In this study, as defined by the U.S. National Centre for Health Statistics (US-NCHS), a chronic disease lasts for a year or longer and is permanent. It results in residual disability, is caused by an irreversible pathological alteration, necessitates specialized training for the patient during rehabilitation, or the patient may need a prolonged period of care, supervision, or observation (44). The primary diagnosis of the patients was categorized according to the International Classification of Diseases (ICD) tenth edition code. It includes chronic endocrine diseases like diabetes and thyroid disorders; chronic circulatory diseases, including hypertension and heart failure; chronic respiratory diseases like asthma and COPD; chronic gastrointestinal diseases including, pancreatitis and liver diseases; and chronic musculoskeletal disorders, including rheumatoid diseases.

#### Charlson comorbidity index (CCI)

In this study, it indicates a severity of a combined comorbidities in patietns with multimorbidity. Three classes were established for the severity of comorbidity based on the CCI score: mild (CCI scores 1–2), moderate (CCI scores 3–4), and severe (CCI scores ≥5) (45).

#### Multimorbidity

It implies the presence of at least two chronic diseases in one individual at the same time (2).

#### Medication adherence

Defined as patients taking their medications as prescribed. The level of medication adherence was measured based on the Adherence in Chronic Diseases Scale (ACDS) and categorized as low adherence if a total score is <21 points; a total score of 21–26 points, medium adherence; and high adherence if a total score > 26 points (46).

#### Medication regimen complexity (MRC)

It indicates the patient’s drug regimen, beyond just the number of medications, which includes the dosage forms, frequency, and instructions for each medication administered (47).

#### Medication regimen complexity index (MRCI)

In this study, the MRCI was defined as the overall patient-level MRCI, including both prescription and OTC medications (48).

#### Polypharmacy

Defined as, when greater than or equal to 5 drugs are prescribed for the patient (39). Polypharmacy did not incorporate the dosage forms, frequency, and instructions for each medications administered.

### Data collection instruments and procedures

A semi-structured data collection instrument was used to gather the data. The instrument was developed by reviewing previous similar studies with some modifications, focusing on the nature of the study population (32, 33). The data were collected through patient interviews, a review of the patients’ medical records, and direct observation. Certain variables were collected through firsthand observation, particularly focusing on the instructions provided for each medication during administration. We directly requested and observed clients as they demonstrated the administration process for special drug formulations that necessitate specific attention and instructions, such as aerosols, eye drops, and suspensions.

The patients’ medical records, including prescription and non-prescription medications, were carefully reviewed and observed. The instrument consisted of three sections. The first section consisted of participants’ sociodemographic and clinical characteristics. In our study, we collected data on patients’ medical history, including their diagnosis, through patient medical record reviews. We ensured that the diagnoses were made by qualified healthcare professionals, such as physicians and specialists, and were recorded in the patients’ medical records. For each disease included in our study, healthcare professionals used standard diagnostic criteria, as established by local, national and international guidelines.

The second section of the instrument consisted of the types of medications used by the patients, and it was used to measure MRC, the MRCI instrument, which included medications’ dosage forms, dosage frequency, and additional instructions. The last section of the data collection instrument consisted of the Adherence in Chronic Diseases Scale (ACDS), a 7-question questionnaire used to measure and categorize the level of medication adherence of the study participants.

As the different diseases vary in terms of their impact on health, each medical condition should objectively be weighed to measure the comorbidity burden using the Charlson Comorbidity Index. The Charlson comorbidity index (CCI) is currently the most commonly used comorbidity assessment tool. It consists of three parts: disease assessment, severity assessment, and scoring. It contains 18 scoring items which including’s warfarin from the drug class. Additional chronic diseases such as cancer, diabetes mellitus, heart attacks, and various other medical conditions were also assessed and scored.

### Outcome measures

This study has two primary outcomes. To measure the levels of MRC in patients with multimorbidity and to examine the association between MRC and levels of medication adherence. Additionally, the study explored factors beyond MRC that influence medication adherence in patients with multimorbidity as a secondary outcome.

The level of MRC was measured by the MRCI instrument, a 65-item validated tool that considers the number of medications, dosage form, dosage frequency, and extra directions (e.g., break/crush the tablet, take at a specified time, and relation to food/liquid). The instrument consists of three sections related to the route of drug administration (section A), dosing frequency (section B), and additional directions (section C). The sum of these sections (A + B + C) contributes to the patient-level MRCI. The MRCI score was categorized into low (≤4), moderate (5–8), or high (>8) levels of MRCI (32, 48). Patient-level MRCI was calculated using the Microsoft Access 2013 electronic data capture tool.

Medication adherence was assessed using the Adherence in Chronic Diseases Scale (ACDS), a 7-question questionnaire. Questions 1–5 addressed the patient’s medication behavior, while 6–7 assessed the doctor-patient relationship. Each item received 0–4 points, with total scores ranging from 0 (minimum adherence) to 28 (maximum adherence). Scores were categorized as high (>26), medium (21–26), or low (<21) (46).

The association between the level of MRCI and medication adherence levels was examined using Pearson’s chi-square test, and further analyzed by ordinal logistic regression to show the impact of MRC on medication adherence.

### Data quality conrtol

Initially, the questionnaire was pre-tested on 21 patients (5%) before the actual data collection and avoided in the final analysis to check clarity, ease of understanding, and cleanliness. The reliability (internal consistency) of the questionnaires was assessed by independent expert personnel in the area. In addition, the Cronbach’s alpha value of the medication regimen complexity measurement tool (*α* = 0.87) and the adherence level measurement tool (*α* = 0.845) were examined and found to be in acceptable ranges. After some modifications to its appropriateness and suitability, actual data collection was followed. Data collectors (two pharmacists and two bachelor nurses) were recruited voluntarily based on their educational level and possible familiarity with medical and health research. They were trained intensively by the principal investigator on the contents of the questionnaire, data collection methods, and ethical concerns. The filled-out questionnaire was checked daily for completeness, clarity, cleanliness, uniformity, and understandability.

### Data management and analysis

Collected data were sorted, cleaned, coded, and entered into Epi-data version 4.6.02 and then exported to SPSS version 26 for analysis. Descriptive statistics like frequencies, means, and percentages were used to summarize categorical and continuous variables. Pearson’s chi-square test was used to assess the association between MRCI levels and medication adherence. Associations of MRC and other associated variables with the level of medication adherence were examined using ordinal logistic regression, given the ordinal nature of the medication adherence levels (low, moderate, and high). The proportional odds (PO) assumption was checked using a likelihood ratio test (*p*-value <0.05) to ensure model suitability. Variables with *p*-values ≤0.25 in the bivariable analysis were included in the multivariable proportional odds model. Adjusted odds ratios (AOR) with 95% CI and *p*-values <0.05 were considered statistically significant.

### Ethical considerations

The proposal was reviewed and approved by the ethical review committee of the Department of Clinical Pharmacy, and the study was ethically approved by the ethical review board of the University of Gondar, with a reference number of UOG-Sop/129/2021. Permission to conduct the study was obtained from the UoGCSH. All individuals enrolled in the study were provided with a written document containing details about the study prior to data collection. For participants who were unable to read or write, the interviewer read out the information sheet and assisted them in providing their signature, typically through a thumbprint. They were also advised to withdraw at any time if they did not want to take part in it. Confidentiality was ensured by making all information anonymous. The data collection procedure was carried out based on the Helsinki Declaration.

## Results

### Sociodemographic and clinical characteristics

Out of a total of 422 participants, 416 (96.8%) participated in the study. The mean (±SD) age was 56.1 (±13.8) (range: 18 to 92) years. Around two-thirds (64.2%, 267) of them were females. Most of the participants (65.9%, 274) had health insurance to cover their medical expenses. Among the study participants, more than half (57.7%, 240) had a diagnosis and had been taking treatments for less than 5 years. More than half of the patients (52.7%, 215) had two chronic conditions. Most of the patients were diagnosed with diseases of the circulatory system (93.5%, 388) (Table 1).

**Table 1:** Sociodemographic and clinical characteristics of study participants attending chronic care follow-up at UoGCSH, Ethiopia, 2021 (*N* = 416).

Variable	Category	Frequency (%)	Mean (±SD)
Sex	Male	149 (35.9)	
	Female	267 (64.2)	
Age	18–29	14 (3.4)	56.1 (±13.8)
	30–39	27 (6.5)	
	40–49	85 (30.4)	
	50–64	159 (38.2)	
	> + 65	131 (31.5)	
Marital status	Single	22 (5.3)	
	Married	317 (76.2)	
	Divorced	54 (13)	
	Widowed	23 (5.5)	
Religion	Orthodox	347 (83.4)	
	Protestant	10 (2.4)	
	Muslim	58 (13.9)	
	Catholic	1 (0.2)	
Educational status	No formal education	117 (28.1)	
	Primary education	134 (32.2)	
	Secondary	87 (20.9)	
	College and above	78 (18.8)	
Occupation	Farmer	63 (15.1)	
	Employed	93 (22.4)	
	Merchant	70 (16.8)	
	Housewife	166 (39.9)	
	Retired	24 (5.8)	
Monthly income in Ehiopian birr (ETB)	<1,500 ETB	128 (30.8)	4765.4 (±1105.3)
	1,500–2,999 ETB	154 (37)	
	3,000–5,000 ETB	80 (19.2)	
	>5000ETB	54 (13)	
Alcohol consumption	Yes	107 (25.7)	
	No	309 (74.3)	
Cigarette smoking	Yes	9 (2.2)	
	No	407 (97.8)	
Residence	Rural	143 (34.4)	
	Urban	273 (65.6)	
Herbal drug use	Yes	62 (14.9)	
	No	354 (85.1)	
Physical exercise	Yes	53 (12.7)	
	No	363 (87.3)	
Source of medication fee	Health insurance	274 (65.9)	
	Out of pocket	142 (34.1)	
Medication administration	Self (autonomous)	259 (62.3)	
	Relatives (required assistance)	157 (37.7)	
Duration of diagnosis in years	< 5	240 (57.7)	4.7 (±3.1)
	5–10	148 (35.6)	
	>10	28 (6.7)	
Duration of treatment in years	< 5	240 (57.7)	4.8 (±2.7)
	5–10	153 (56.8)	
	>10	28 (6.7)	
Previous hospitalization	No	149 (35.8)	
	Yes	267 (64.2)	
Number of drugs per patient	<5	223 (53.6)	4.8 (±2.3)
	≥5	193 (46.4)	
Number of Multimorbidity	2	215 (51.7)	2.6 (±1.7)
	3	158 (38)	
	≥ 4	43 (10.3)	
Charlson comorbidity index (CCI)	Mild	328 (78.8)	
	Moderate	56 (13.5)	
	Severe	32 (7.7)	
*Primary diagnosis of study participants	Disease of the Circulatory system	388 (93.4)	
	Disease of the endocrine system	220 (53.0)	
	Disease of respiratory system	57 (13.7)	
	Disease of the renal system	27 (6.5)	
	Disease of the gastrointestinal system	21 (5)	
	Disease of hematology system	17 (4.1)	
	Disease of the central nerve system	16 (3.8)	
	Disease of the musculoskeletal system	12 (2.9)	
	Others	24 (5.9)	

* one patient could have diseases of different systems, Others, diseases of the eye and adnexa, diseases of the skin and subcutaneous tissue, neoplasms, mental and behavioral disorders, nutritional and electrolyte disorders.

### Medication regimen complexity and medication adherence

**Figure 1:**
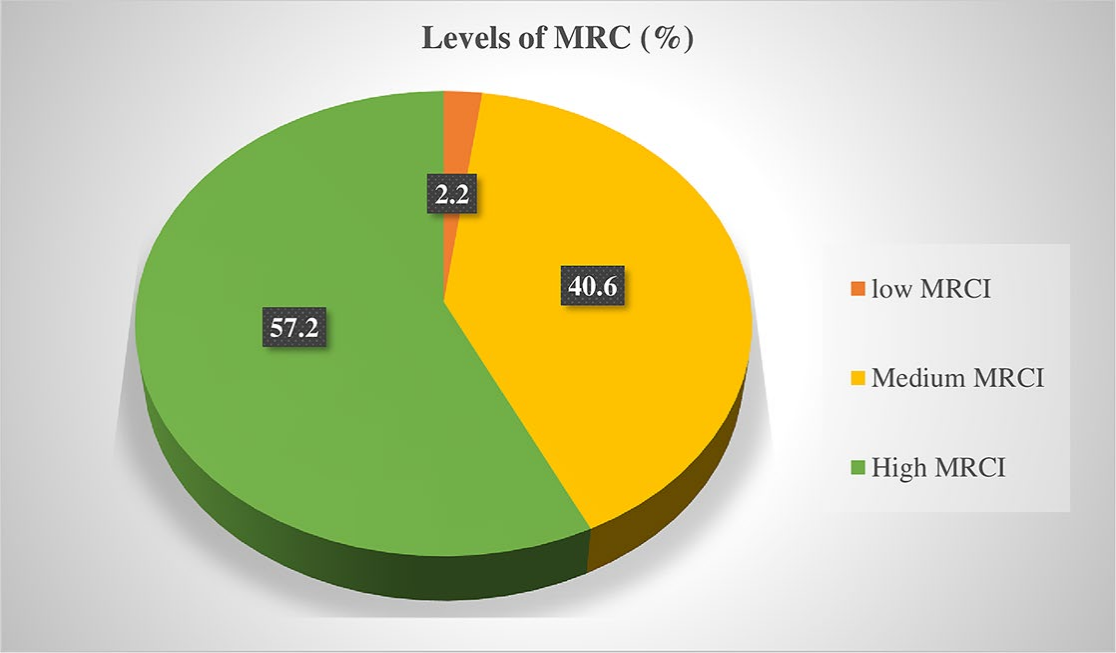
Level of MRC in patients with multimorbidity at the UoGCSH, 2021.

The total number of medications used for the management of studied patients were 1980. Commonly prescribed drug classes were cardiovascular drugs (32.6%, 645) followed by endocrine drugs (18.2%, 360). Almost half of the patients (46.4%) had been taking ≥5 drugs, with a mean (±SD) medication count per patient of 4.8 (±2.3). The MRCI score ranged from 2 to 20, with an average score of 9.7 (±3.4). Almost half of patients (49.3%) had low medication adherence (Table 2). The majority of patients (57.2%, 237) with a 95% CI (52.4, 61.8) were found to have a high complexity score (Figure 1).

**Table 2:** Distribution of medications used by patients, medication complexity score, and level of medication adherence.

Variables	Category	Frequency (%)	Mean (±SD)
Class of medications (*N* = 1980)	Cardiovascular drugs	645 (32.6)	
	Endocrine drugs	360 (18.2)	
	Respiratory drugs	265 (13.4)	
	Analgesic and anti-pyretic	196 (9.9)	
	Gastrointestinal drugs	173 (8.7)	
	Hematology Drugs	132 (6.7)	
	Centeral nerve system drugs	84 (4.2)	
	Others	125 (6.3)	
Medication count per patient (*N* = 416)	< 5	223 (53.4)	4.8 (±2.3)
	≥5	193 (46.6)	
Avrage MRCI score	-		9.7 (±3.4)
Level of medication adherence (*N* = 416)	Low adherence	205 (49.3)	
	Moderate adherence	125 (30)	
	High adherence	86 (21)	

Others, Chemotherapy (antibiotic), musculoskeletal drugs, vitamin and mineral drugs.

### Association between MRCI and medication adherence level

Pearson’s chi-square test of association was used to identify a statistically significant association between MRCI and level of adherence, and the results revealed that patients with a higher MRCI had lower medication adherence (*p* = 0.003) (Table 3).

**Table 3:** Association of MRCI and levels of medication adherence using Pearsno’s chi-square test in patients with multimorbidity at the UoGCSH.

Level of medication adherence (*n*, %)						*p*-value
		Low	Moderate	High	Total	
MRCI levels	Low	4 (1)	2 (0.5)	3 (0.7)	9 (2.2)	0.003
	Medium	73 (17.5)	46 (11.1)	50 (12.0)	169 (40.6)	
	High	128 (30.1)	77 (18.5)	33 (7.9)	238 (57.2)	

### Factors associated with medication adherence

The multivariable ordinal logistic regression analysis showed that partcipants’ income level, duration of follow-up, severity of CCI, number of medications, and MRCI levels were significantly associated with medication adherence. The odds of having higher medication adherence in individuals with a monthly income of >5,000 were increased by 4.6 times (AOR = 4.59, 95% CI: 2.14, 9.83) compared with those who had a monthly income level of <1,500 Ethiopian birr. Simultaneously, the odds of medication adherence were higher in patients who had >10 years of follow-up duration compared with patients who had <5 years of follow-up duration (AOR = 2.31, 95% CI: 1.10, 4.87). On the other hand, the odds of medication adherence among individuals who had severe CCI decreased by 64% (AOR = 0.36, 95% CI: 0.15, 0.83) compared with those patients with mild CCI. Patients who were on polypharmacy (≥5 medications) were also found to have lower odds of medication adherence compared with those who received <5 medications (AOR = 0.63, 95% CI: 0.41, 0.97). More importantly, patients with a medium (AOR = 0.43, 95% CI: 0.04, 0.72) and a high (AOR = 0.31, 95% CI: 0.07, 0.79) MRCI level were found to have decreased odds of medication adherence compared to patients with a low level of MRCI levels (Table 4).

**Table 4:** Assocaition between independent variables and medication adherence in patients with multimorbidity at the UoGCSH.

Variables		COR (95% CI)	*p*-value	AOR (95% CI)	*p*-value
Gender	Male	1		1	0.097
	Female	1.79 (1.22, 2.63)	0.003	1.45 (0.93, 2.25)	
Level of education	No formal education	1		1	
	Primary education	1.26 (0.74, 2.14)	0.391	0.85 (0.45, 1.59)	0.641
	Secondary	1.44 (0.87, 2.57)	0.22	1.1 (0.60, 2.12)	0.715
	College and above	1.05 (0.615, 1.841)	0.843	1.02 (0.43, 2.74)	0.183
Residence	Urban	1		1	0.355
	Rural	0.67 (0.45, 0.98)	0.04	0.80 (0.50, 1.27)	
Monthly Income level	<1,500	1		1	
	1,500–2,999	2.38 (1.667, 6.0720)	0.045	1.71 (0.63, 6.77)	0.135
	3,000–5,000	2.25 (0.87, 4.56)	0.065	1.56 (0.43, 5.76)	0.321
	>5,000	3.5 (1.86, 6.67)	<0.001	4.59 (2.14, 9.83)	0.011*
Alcohol history	No	1		1	0.346
	Yes	0.64 (0.42, 0.98)	0.042	0.79 (0.4, 1.27)	
Traditional medicine use	No	1		1	0.417
	Yes	0.67 (0.39, 1.15)	0.144	0.79 (0.45, 1.39)	
Cost of healthcare service	Health insurance	1		1	0.277
	Out of pocket	0.73 (0.4, 1.06)	0.101	0.7 (0.511, 1.21)	
Managing medicine	Autonomous	1		1	0.142
	Required assistance	0.61 (0.41, 0.89)	0.010	0.73 (0.48, 1.12)	
Duration of follow-up in years	< 5	1		1	
	5–10	1.07 (0.73, 1.59)	0.707	1.11 (0.729, 1.70)	0.613
	>10	1.83 (0.95, 3.55)	0.078	2.31 (1.0, 4.86)	0.027*
Leve of CCI	Mild	1		1	
	Moderate	0.81 (0.47, 1.40)	0.453	0.88 (0.49, 1.56)	0.679
	Severe	0.37 (0.17, 0.80)	0.012	0.36 (0.15, 0.82)	0.016*
Number of medications	< 5	1	0.021	1	0.036*
	≥ 5	0.48 (0.33, 0.7)		0.63 (0.41, 0.97)	
MRCI levels	Low	1		1	
	Medium	0.39 (0.02, 0.85)		0.43 (0.04, 0.72)	0.015*
	High	0.30 (0.06, 0.82)		0.31 (0.07, 0.79)	0.001*

CCI, Charlson comorbidity index; ^*^ statistically significant at *p* < 0.05.

## Discussion

To the best of the investigators’ knowledge, this is the first study that assessed MRC and its association with medication adherence that accounts for the complexity of medication in patients with multimorbidity in Ethiopia. In the current study, MRCI scores were stratified by low, medium, and high regimen complexity. The majority of participants had a high MRCI score. The finding showed a significant association: patients with higher MRCI levels were more likely to have lower odds of high medication adherence. In addition to MRC, monthly income, duration of follow-up, number of medications, and CCI levels were significantly associated with medication adherence in patients with multimorbidity.

Consistent with earlier studies, a current study also showed that most patients were found to have a higher MRCI score with a higher medication count (49, 50). This might be because patients with multimorbidity could have prescriptions for multiple medications, which is responsible for the complexity of their regimens. The complexity of pharmacotherapy is composed of multiple features of the prescribed regimen, including the number of different drugs in the treatment, the number of doses of each drug per day, the number of per-dose dosage units, the total number of doses per day, and drug interactions with food (51). In contrast, the MRCI score in the current finding was higher in the previous study done in Brazil (52). This discrepancy may be due to the use of different cut-off points, the treatment approach of clinicians in line with guidelines, and the difference in the sociodemographics of patients. As compared with previous studies conducted on Ethiopian patients with specific diseases such as asthma (28) and diabetes (32), a higher proportion of patients were found to have a higher MRCI score in the current study. This might be because of the current study conducted on patients with multimorbidity who could have additional prescribed medications because of multiple medical conditions. As a result, a higher level of MRC in patients with multimorbidity may suggest that physicians need to be vigilant to minimize the complexity of medications in such risky patients by applying the deprescription of inappropriate medications and simplification of regimens (53). Identified strategies that were used to reduce the complexity of medication regimens should be considered, like, fixed-dose combination (FDC), once-daily dosing, and a combination of more than one approach. Educating and empowering patients to understand the treatment regimen and its benefits should be considered. More over Work diligently with patients and families to secure an accurate list of medications, Reorganize the medication list,look for inappropriate and incorrect prescriptions, and use caution when deprescribing medications.

Patients with multimorbidity with complex medication regimens are expected to adhere to their medications to be effective in their treatment outcome. However, in this study, the findings revealed that a significant number of participants had low levels of adherence to their medications. Similarly, earlier studies done in the United Arab Emirate (54), Saudi Arabia (55), and South India (56) showed that a significant number of study subjects had a level of low medication adherence. A study done on Ethiopian diabetes patients with comorbidity also showed significantly low medication adherence (15). The findings may implicate that patients with multimorbidity need strict follow-up and support to adhere to their prescribed medications. However, the findings varied with the study done in Spain (57), which revealed that a significant proportion of the study participants were in the range of high levels of medication adherence. This variation potentially might be due to differences in patients’ medication knowledge and perceptions about medication complexity and fear of side effects. The current study was conducted on patients with low levels of medication and health literacy compared with the earlier study. High medication costs in patients with polypharmacy might also be a potential reason for low medication adherence. In addition to this attributed differences in the study setting, methods used to measure medication adherence, and physicians’ and pharmacists’ approach to their patients could bring differences in patients’ attitudes toward their medication.

Although MRC and medication adherence are different outcome measures, it is believed that MRC has been associated with low medication adherence. Consistent with earlier studies conducted on Ethiopian patients with asthma (28) and diabetes (32), the current study also disclosed that patients with a higher level of MRC were found to have significantly lower levels of medication adherence. This is also in line with other studies conducted worldwide (22, 23, 49, 50, 54). This could be because patients with multimorbidity are potentially treated with polypharmacy, which can be the reason for the increased MRC burden, and in turn, it could result in poor medication adherence. An increasing number of patients with multimorbidity means that more and more patients are faced with complex medication regimens. As a result, patients with multimorbidity who have been treated with polypharmacy should be assessed for medication adherence levels. The findings may suggest that as patients with multimorbidity are at high risk of having high MRCI and low levels of medication adherence, due to the presence of multiple chronic comorbidities and polypharmacy for long durations, close follow-up of these patients could be warranted.

The present study has identified the association of participants’ sociodemographic and clinical variables with the level of medication adherence beyond the complexity of medication regimens. Consistently with previous studies (22, 23, 58), the current study showed that monthly income, duration of diseases, CCI levels, and number of medications were found to have a significant association with the level of medication adherence. The associated factors identified in this study were also largely consistent with findings from previous large-scale territory-wide study using patient health records in the Chinese population (59) and Italian (60). The findings may implicate that most of the independent factors that affect medication adherence in patients with multiple chronic diseases are similar and need interventions to enhance the treatment outcome of patients. On the other hand, participants’ age, level of education, and social drug use were also associated with the level of medication adherence in previous studies (55, 61–63). The difference could be due to variability in the study participants, adherence measurement tools used, healthcare systems and policies, and knowledge, skill, and patient care approaches of healthcare professionals. In addition, we need to consider that heterogeneity of factors related to diseases may affect the patient’s medication-taking behavior. For example, the treatment approach of clinicians and patients’ lifestyles, medication-related knowledge, and perceptions may have a pivotal impact on variability.

Consistently with earlier studies (15, 64–66), the current study showed that patients with a lower monthly income were found to have lower odds of high medication adherence. This could be because patients with low economic status and household income have the potential to withdraw medications because of affordability issues. This problem might be severe in chronic illnesses and patients with comorbidities because of increased medication costs for treating additional conditions. Particularly in Ethiopian settings, most patients are of low socioeconomic status (67, 68). On the contrary, most patients with chronic diseases have comorbid conditions and receive multiple medications (36–39). Thus, patients with a lower income will have a lower level of medication adherence because most multimorbidity patients may be unable to afford treatment costs for multiple medications. Therefore, the findings may suggest that healthcare practitioners and prescribers should devise strategies to acknowledge patients’ socioeconomic situation and facilitate effective and transparent communication regarding the pricing of prescribed medications. Patients may also benefit from engaging in Ethiopian community-based health insurance (CBHI) programs (69), which may help individuals by providing optimum pre-paid coverage costs and protecting them from catastrophic expenditures.

In this study, patients treated with several medications (polypharmacy) and a higher Charlson commorbidy index (CCI) were found to be lower odds of medication adherence. The finding is in line with previous studies (22, 23, 32, 49, 50). The association might be because patients with polypharmacy and a higher number of comorbidities may have poor medication adherence due to the complexity of medication regimens, potential adverse effects related to potential drug–drug interactions, and the affordability issue of multiple medications. The loss of medication administration time may also be caused by taking more medications. Healthcare professionals, especially prescribers, should thus concentrate on honing the art of writing prescriptions for the least amount of pharmaceuticals possible while taking into account the requirement for medication treatment for patients with multimorbidity. To manage potential and existing comorbidities, patients also need to be extremely watchful and driven to take all of their prescribed medications.

In addition, the possible reason associated with low level of adherence was due to those with multiple medical conditions might encounter difficulties to complying with their daily medication-taking schedule, probably due to poorer bodily function and the higher possibility of polypharmacy, referrals or adverse drug events. The CCI score highlights the severity of chronic disease and chronicity which affects self-managing to take medications. Moreover, CCI indicated the severity of the disease status, which results difficulty of taking medications regularly and autonomously.

In general, the current study finds that patients with multimorbidity have a high prevalence of MRC, which affects medication adherence negatively. The numerous barriers to the effective use of medications in resource-limited settings include poor communication between the patient and physician, inappropriate knowledge gaps on medications, fears of adverse events, long-term therapy, polypharmacy, and cost and access barriers (70). These barriers could have a significant impact, particularly on patients with multimorbidity receiving complex medication regimens. Therefore, the identification of specific barriers for each patient and the design of appropriate prevention strategies are indispensable to mitigating medication adherence.

## Strengths and limitations of the study

Considering the burden of patients with multimorbidity, the present study has highlighted the level of MRCI and medication adherence, which has a significant implication for further study. However, there are some limitations to the present study that need to be considered. First, it is a cross-sectional study design, whereby claims about the directionality of the causal relationship between the dependent and independent variables cannot be verified. Secondly, MRCI was calculated using only what was captured in the patient’s medical chart. As a result, any medications or instructions not recorded were missed. Another limitation was that the extent of generalizability may be limited since it was a single-center study. The authors would like to welcome further studies with prospective follow-up, including a relatively larger population in multicenter settings.

## Implication and contribution to the field

This study provides crucial data from an understudied region, Ethiopia, like many African countries, faces a growing burden of multimorbidity but has limited research on medication adherence and regimen complexity. This article contributes valuable data from this region, informing healthcare strategies and interventions. Investigates a key factor in multimorbidity management that medication regimen complexity is a significant barrier to adherence in multimorbid patients, leading to poorer health outcomes. This study sheds light on the specific challenges faced by Ethiopian patients and the impact on adherence. The findings also inform intervention development by identifying factors associated with low adherence (e.g., high regimen complexity), the study helps tailor interventions to the specific needs of Ethiopian patients with multimorbidity. This could include simplified regimens, medication reminders, or educational programs. It can also raise awareness of patient burden by highlights the challenges faced by multimorbid patients in managing complex medication regimens, urging healthcare professionals to consider patient perspectives and advocate for patient-centered care. The also study opens doors for further research on effective interventions to improve adherence in Ethiopian patients with multimorbidity. This could involve testing different strategies and evaluating their impact on clinical outcomes.

Overall, the article makes a valuable contribution to the field of medicine by providing data and insights on medication adherence in a previously understudied population. This can inform healthcare practices, intervention development, and further research, ultimately improving health outcomes for multimorbid patients in Ethiopia.

## Conclusion

Medication regimen complexity was highly prevalent in patients with multimorbidity and had a significant impact on medication adherence. In addition, patients with low income, a shorter duration of treatment, and those with polypharmacy and a high CCI should strictly follow and require intervention to maximize their medication adherence to enhance treatment outcomes and reduce healthcare costs in patients with multimorbidity. Physicians and healthcare providers could be engaged in strategies to simplify and minimize MRC in patients with multimorbidity.

## Data availability statement

The raw data supporting the conclusions of this article will be made available by the authors, without undue reservation.

## Ethics statement

The studies involving humans were approved by the Ethical Review Committee of the Department of Clinical Pharmacy and the study was ethically approved by the Ethical Review Board of the University of Gondar, with a reference number of UOG-Sop/129/2021. The studies were conducted in accordance with the local legislation and institutional requirements. The participants provided their written informed consent to participate in this study.

## Author contributions

AK: Conceptualization, Data curation, Formal analysis, Funding acquisition, Investigation, Methodology, Project administration, Resources, Writing – original draft, Writing – review & editing. AS: Formal analysis, Investigation, Methodology, Software, Validation, Visualization, Writing – original draft, Writing – review & editing. AM: Formal analysis, Methodology, Software, Supervision, Validation, Visualization, Writing – original draft, Writing – review & editing. BG: Investigation, Methodology, Project administration, Software, Supervision, Validation, Visualization, Writing – original draft, Writing – review & editing.
